# Effect of feeding fermented soybean meal on broiler chickens’ performance: a meta-analysis

**DOI:** 10.5713/ab.21.0546

**Published:** 2022-06-24

**Authors:** Agung Irawan, Adi Ratriyanto, Adib Norma Respati, Niati Ningsih, Rahma Fitriastuti, Wara Pratitis Sabar Suprayogi, Rendi Fathoni Hadi, Wahyu Setyono, Novi Akhirini, Anuraga Jayanegara

**Affiliations:** 1 Vocational School, Universitas Sebelas Maret, Surakarta 57126, Indonesia; 2 Animal Feed and Nutrition Modelling Research Group (AFENUE), Department of Nutrition and Feed Technology, Faculty of Animal Science, IPB University, Bogor 16680, Indonesia; 3 Department of Animal and Rangeland Sciences, Oregon State University, Corvallis 97331, OR, USA; 4 Department of Animal Science, Faculty of Agriculture, Universitas Sebelas Maret, Surakarta 57126 Indonesia; 5 Department of Animal Production, Politeknik Negeri Jember, Jember 68124 Indonesia; 6 Politeknik Pertanian Negeri Pangkajene Kepulauan, South Sulawesi 90761, Indonesia; 7 Department of Nutrition and Feed Technology, Faculty of Animal Science, IPB University, Bogor 16680, Indonesia

**Keywords:** Broiler Chickens, Fermentation, Meta-analysis, Inoculant, Soybean Meal

## Abstract

**Objective:**

The present study aimed to quantify the effects of fermented soybean meal (FSBM) on broiler chickens’ performance by employing a meta-analysis approach.

**Methods:**

A total of 16 studies were included in the database after being systematically selected using a PRISMA protocol. Hedges’ *g* effect size was used to quantify pooled standardized mean difference (SMD) using random-effects models at 95% confidence intervals (95% CI). Publication bias among studies was computed with Egger’s test and visualized using funnel plots.

**Results:**

Results indicated that dietary FSBM inclusion increased final body weight (BW) (SMD = 0.586, 95% CI: 0.221 to 0.951, p = 0.002) of broiler chickens, particularly in starter period (SMD = 0.691, 95% CL: 0.149 to 1.233, p = 0.013) while in the finisher period, the effect was weaker (SMD = 0.509, 95% CI: 0.015 to 1.004, p = 0.043). Average daily gain (ADG), feed intake (FI), and feed conversion ratio (FCR) were not affected with FSBM inclusion when compared to control. Subgroup analysis revealed that FI increased in starter period (SMD = 0.582, 95% CI: 0.037 to 1.128, p = 0.036). When considering types of microorganism as moderating variables in the subgroup analysis, we found that *Aspergillus oryzae*, mixed probiotics+bromelain protease, *Bacillus subtilis*, and *Lactobacillus* bacteria significantly increased ADG and FI (p<0.01). Additionally, either *Bacillus subtilis*+protease or *Bacillus subtilis* alone decreased FCR (p<0.001). However, meta-regression analysis showed that levels of FSBM inclusion had no effects on final BW (p = 0.502), ADG (p = 0.588), FI (p = 0.861), and FCR (p = 0.462).

**Conclusion:**

Substituting SBM in broiler chickens’ diet with FSBM improved BW of broiler chickens, especially in the starter period whereas the effects on ADG, FI, and FCR were mostly dependent on microbial strains used for fermentation.

## INTRODUCTION

Soybean meal (SBM) is a major contributor of protein and indispensable amino acids such as lysine, tryptophan, threonine, isoleucine, and valine in broiler diets [1]. These nutritional components play a crucial role to support the rapid metabolism and growth rate of modern broiler chickens. However, the presence of anti-nutritional factors (ANFs) such as protease inhibitor (i.e. trypsin inhibitor), antigenic protein, and phytic acid limits the nutrients’ bioavailability and digestion thus impairing the growth of broilers and their health status [2,3]. A considerable amount of undigested protein that enters the broiler hindgut is detrimental. The undigested protein can form toxic compounds such as ammonia and polyamine and it becomes fermentable for bacterial pathogens [3,4]. An excessive amount of fermentable proteins can promote an increase of pathogenic bacteria associated with the incidence of intestinal diseases such as coccidiosis and necrotic enteritis (NE) on farm [5].

In the last two decades, microbial fermentation has been proposed as an economically feasible method to enhance the nutritional content as well as eliminate the ANFs concentration of SBM [2,6,7]. Fermented-SBM (FSBM) is a product resulted from fungal or bacterial fermentation (mainly *Aspergillus niger*, *Aspergillus oryzae*, *Bacillus subtilis*, and *Lactobacillus* group) [8–11]. Previous studies reported that fermentation successfully decreased ANFs and increased protein and amino acids composition of SBM. For instance, Wu et al [7] reported considerable increase of threonine, leucine, and isoleucine by 29.8%, 34.7%, and 27.8%, respectively, by fermenting SBM with *Bacillus stearothermophilus* (ATCC 7953). Microbial fermentation of *bacillus*, l*actobacillus*, and yeast was also reported to increase protein composition of SBM from 8.2% to 18.9% [6,10] as well as removed antinutrient substances and enhanced nutrient utilization [2,12]. These beneficial effects led in an improvement of broiler growth performance and feed efficiency.

There is accumulating evidence of significantly increased average body weight gain (BWG), final body weight (BW), and feed efficiency [6,11,13]. Other reports also suggested that FSBM positively modulated ileal and cecal microbial composition, intestinal morphology, immune status, and carcass traits of broiler chickens [6,7,11,14]. However, inconsistent results were observed whereas several studies with FSBM inclusion did not show any effect on average daily gain (ADG) and final BW [13,15]. There was also an indication of level-dependency in which a decreasing trend on BW was detected in studies that used a used high level of FSBM [16,17]. In the experiments, FSBM was used as either total or partial replacement to SBM which may contribute to the magnitude of the effect on broiler performance and feed efficiency. Inclusion of FSBM varied among studies, ranging from less than 2% to 30% of the diet [13,15].

To our knowledge, there is no consensus regarding the threshold of FSBM inclusion in the diet. However, large variation in the inclusion levels, microbial types used as fermenters, and environmental conditions among experiments might elucidate why the outcomes differed. Thus, we hypothesized that levels of FSBM and microbial fermenters are two major factors contributing to the effect size on broiler growth performance. To generalize the effect, it is useful to integrate available studies and calculate the effect size with relevant statistical method [18]. The present study therefore aimed to quantify the effect of FSBM on broiler performance and feed efficiency by employing a meta-analysis approach.

## MATERIALS AND METHODS

### Literature search and selection criteria

A literature search was performed on scientific digital platforms of Science Direct ( https://www.sciencedirect.com), PubMed Central ( https://www.ncbi.nlm.nih.gov/pmc/), and Google Scholar ( https://scholar.google.com) with the keywords “fermented”, “soybean meal”, and “broiler chicken”. In the PubMed central, we used combinatory terms with specific Boolean operators as follows: ((fermented [All Fields] AND ("soybeans"[MeSH Terms] OR "soybeans"[All Fields] OR "soybean"[All Fields]) AND ("meals"[MeSH Terms] OR "meals"[All Fields] OR "meal"[All Fields])) AND (broiler [All Fields] AND ("chickens"[MeSH Terms] OR "chickens"[All Fields])). All relevant titles of articles from the platforms were imported into the reference manager to easily select the papers.

Preferred reporting items for systematic review and meta-analysis (PRISMA) protocol was employed to reduce publication bias and to ensure the quality of meta-analysis [19]. Accordingly, inclusion criteria were determined as follows: i) the article was published in English in a peer-reviewed journal; ii) reported FSBM inclusion level in the diet, processing method as well as microbes used as fermenter; iii) described the broiler strain, length of rearing periods, replication, and sample size per replicate (n); iv) used similar nutrient specification in the treatment and control groups; v) provided means and appropriate variances (standard deviation or standard error of the means) for at least the variables of BW or feed intake (FI). Experiments reporting only ADG or feed conversion ratio (FCR) were considered because they allowed to calculate final BW and FI, and vice versa. In addition to the criteria, studies that used soy protein concentrate, probiotics, or prebiotics (i.e. mannan-oligosaccharide) were not included because these materials may interfere the results. Challenged experiments with pathogens were also disregarded.

**Figure 1:**
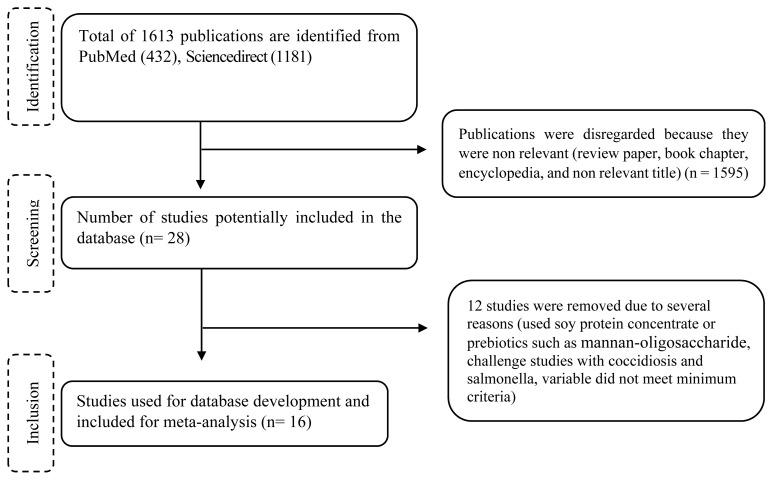
Flow charts of selection process of literature used for the meta-analysis.

In total, 1,613 articles were detected by employing the above-mentioned strategy. A total of 365 papers were excluded because they were review articles (86), book chapters (147), Encyclopedia (23), and other non-relevant types of articles (109). Following this, 1,248 remaining papers were evaluated to meet the criteria. Two investigators (Niati Ningsih [NN] and Rahma Fitriastuti [RF]) reviewed the titles and abstracts of the papers and excluded non relevant publications from the imported database. Subsequently, two independent investigators (Agung Irawan [AI] and Adib Norma Respati [ANR]) reviewed the selected studies to assess the risk of publication bias among individual studies [20,21]. In the subsequent step, the investigators (NN and RF) extracted data from the selected articles and did calculations for analyses. In total, 16 studies representing 68 comparisons were eligible and therefore were integrated into the database (Table 1). The selection process of the studies is provided in Figure 1.

### Data extraction

Information on the broiler chickens used (number, strain, sex), replication, study design, FSBM inclusion level including processing methods and microbes used for fermentation, country in which the experiment was carried out, and nutrient compositions of the diet in respective periods (starter, grower, finisher) such as metabolizable energy (ME), crude protein (CP), lysine, and methionine were retrieved from each paper into Microsoft Excel spreadsheet. The main outcomes used for meta-analysis were final BW (g), ADG (g/d), FI (g/bird/period), and FCR (g feed/g BW). Therefore, mean values including the corresponding variant (standard deviation [SD]) or standard error of the mean (SEM) of each unit of comparison were extracted. The SD value of each study was calculated from SEM using the formula: SD = SE×sqrt(n); where n = sample size or number of replicates, when the study only provided SE rather than SD value. When available, the values from graphical data were extracted by employing an extraction tool of WebPlotDigitizer ( https://apps.automeris.io/wpd/). The summary of the studies used for meta-analysis is presented in Table 1 while summary statistics of the nutrient specification and outcome variables are shown in Table 2.

### Statistical analysis

Meta-analysis was performed using the *meta* and *metafor* packages [26] available in R statistical software. Means of control and treatment in the studies were considered as continuous outcome data. Effect size of individual study was calculated with Hedges’ *g* and expressed as standardized mean differences (SMD) in a forest plot following a random-effects model at 95% confidence intervals (CIs). We considered Hedges’ *g* because it is easy to interpret and has strong statistical power which can control study bias that may occur in small sample sizes [27]. Hedges’ *g* outcome estimates >0 with p≤0.05 show a significant higher on BW, ADG, FI, or FCR due to FSBM inclusion when compared to control. Heterogeneity index (*I*^2^) among studies was analyzed using the DerSimonian and the Laird test (*Q*-statistic) at a significance level p≤0.05. The degree of heterogeneity was categorized as no heterogeneity (0<*I*^2^≤25%), low (25%<*I*^2^≤50%), moderate (50%<*I*^2^≤75%), and high (*I*^2^>75%) [28].

Meta-regression was conducted to evaluate the relationship between FSBM inclusion levels with the effect size (Hedges’ *g*) of the outcome variables. In addition, subgroup analysis was also performed to quantify the effect sizes of several factors that may influence the magnitude of the effect size. In this study, rearing period (starter and finisher) and type of microbes used to ferment SBM were used in the subgroup meta-analysis using random effects models.

**Figure 2:**
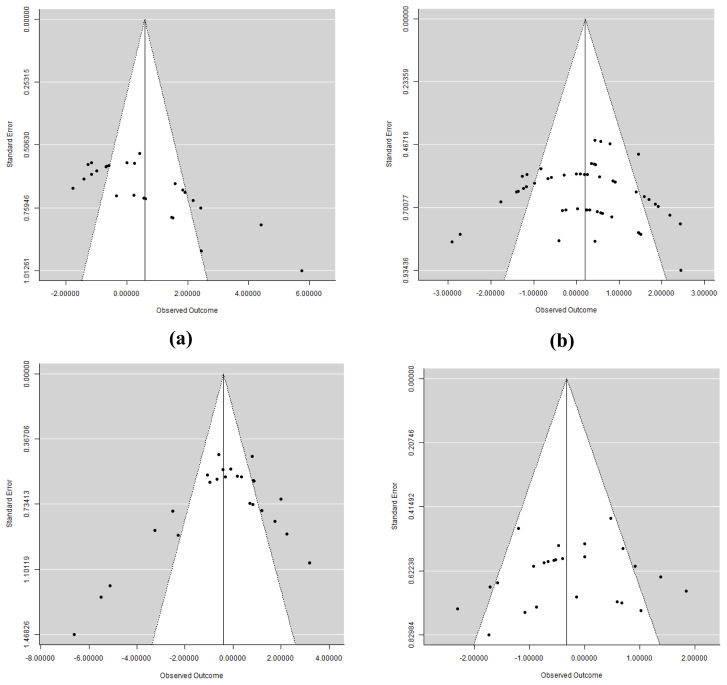
Funnel plots analysis on (a) body weight, (b) average daily gain, (c) feed intake, and (d) feed conversion ratio to detect publication bias between-study.

In addition to the risk of bias from studies, publication bias was assessed by using funnel plots and the results are presented in Figure 2. Egger’s test was conducted to know the presence of bias [29]. Bias among publications was considered to be significant at p≤0.05. In case there was a presence of bias, sensitivity analysis was carried out using the leave-one-out study analysis to compare between-study heterogeneity (*I*^2^) [28] and to determine the effect size of the studies contributing to the bias [30].

## RESULTS

### Description of the studies included in the meta-analyzes

This study included 5,176 broiler chickens from various strains predominantly by Ross 308 (57.14%) while the rest were Arbor Acres (21.43%), Cobb 500 (14.29%), and Vencob (7.14%). A total of 35.71% of studies used either male or mixed sexes of broiler chickens while others were female (14.30%) and not given (14.30%). All studies used corn and soybean-based diet for their experiments. Inclusion levels of FSBM varied among studies, from 15 g/kg diet to 361 g/kg diet (Table 1). In each study, microbial strain for SBM fermentation also varied in which *Bacillus subtilis* was the most frequently used, corresponded to 57.14% of the studies, either in a single strain or mixed with other microorganisms. Other microorganisms applied for fermentation were fungi (*Aspergillus niger* and *Aspergillus oryzae*), yeast (*Saccharomyces cerevisiae*), and from lactic acid bacteria group such as *Bacillus subtilis*, *Lactobacillus* spp., *Lactobacillus acidophilus*, *Lactobacillus plantarum*, and *Bacillus stearothermophilus*. Information on the nutrient composition of each study was summarized and is presented in Table 2 for ME (kcal/kg), CP (%), lysine (%), and methionine (%) both in starter and finisher phases. As indicated in Table 2, the nutritional specifications were appropriate to the nutrient recommendation of NRC [31]. Additionally, descriptive statistic of the chemical compositions of SBM and FSBM are provided in Table 3. Processing SBM to FSBM increased CP by 14.3% while decreased trypsin inhibitor, Glycinin, mg/g, and β-Conglycinin, mg/g by 83.1%, 82.2%, and 79.7%, respectively.

### Effect of fermented soybean meal on body weight

Summary of meta-analysis is presented in Table 4 and the forest plots for subgroup analysis are presented in the Supplementary materials 1. Comparisons between studies with or without FSBM in the present meta-analysis suggested that replacing SBM with FSBM had a significant positive effect on final BW of broiler chickens as shown from the pooled SMD (SMD = 0.586, 95% CI: 0.221 to 0.951, p = 0.002), regardless of inclusion levels. Since there were high variations of between-study heterogeneity (83.5% of *I*^2^ statistic), subgroup analyzes were performed to indicate which factors mediating the effect sizes. Results from subgroup analysis based on rearing period showed that significant increase was more pronounced in the starter period (≤21 d) (SMD = 0.691, 95% CL: 0.149 to 1.233, p = 0.013) compared to finisher period (>21) (SMD = 0.509, 95% CI: 0.015 to 1.004, p = 0.043) with considerable between-study variations (84.9% and 82.2% of *I*^2^ statistics, respectively). In addition, most microbial fermentation resulted in a significant increase of final BW except for *Aspergillus niger* (SMD = 0.259, 95% CI: −0.322 to 0.839, p = 0.382) and multiple strain microbes containing lactic acid bacteria and yeast (SMD = 0.191, 95% CI: −0.274 to 0.656, p = 0.420).

### Effect of fermented soybean meal on average daily gain

Results from the Egger’s test indicated that there was no publication bias (p>0.05) for BW, FI, and FCR among studies which was supported by symmetrical funnel plots (Figure 2). However, there was a significant publication bias on ADG (p = 0.047). Thus, we performed a sensitivity analysis to detect source of bias. As a result, Wang et al [32] was eliminated from the dataset because it had the highest effect size among others and contributed to increase the heterogeneity index of the outcome. After excluding Wang et al [32] as the major source of variance, there was an improvement in publication bias (p = 0.069) and therefore we decided to use this final dataset for meta-analysis. However, there was no different on the SMD before and after removing the data of Wang et al [32]. Forest plots of subgroup analysis on FI are given in the Supplementary Figures 1 to 8. Overall SMD of ADG as a result of new dataset was 0.294 (95% CI = −0.060 to 0.648, p>0.10) with high heterogeneity (*I*^2^ = 66.3%, *Q*<0.001). Although there was no effect both in starter and finisher period, however, positive effects were detected corresponding to the FSBM with *Aspergillus oryzae* (SMD = 0.258, 95% CI: −0.322 to 0.838, p = 0.002), Mixed probiotics+bromelain (SMD = 0.612, 95% CI: 0.027 to 1.196, p<0.001), *Bacillus subtilis* (SMD = 1.640, 95% CI: 0.712 to 2.567, p = 0.006), and *Lactobacillus* microbes (SMD = 0.591, 95% CI: 0.172 to 1.009, p<0.001) (Table 4) in which no heterogeneity was detected (*I*^2^ = 0.00%, *Q*>0.10). By contrast, SBM fermented with *Bacillus stearothermophilus* significantly decreased the ADG (SMD = −1.654, 95% CI: −2.323 to −0.985, p<0.001) with low level of heterogeneity (*I*^2^ = 32.6%, *Q* = 0.191).

### Effect of fermented soybean meal on feed intake

Of the 51 comparisons satisfying the inclusion criteria, treatment with FSBM did not alter FI (SMD = 0.115, 95% CI: −0.272 to 0.502, p = 0.560) at the degree of heterogeneity of 85.9% (*Q* = 0.001) (Table 4). However, when it turned to rearing period, FSBM utilization significantly increased the FI in the starter period (SMD = 0.582, 95% CI: 0.037 to 1.128, p = 0.036) with high heterogeneity (*I*^2^ = 80.3%, *Q* = 0.001), but it did not affect FI in finisher period (p = 0.223). Subgroup analysis on microbes used in the fermentation process indicated that groups of *Aspergillus oryzae*, *Bacillus subtilis* +protease, and mixed of probiotics+bromelain were responsible for increasing the FI. Contrary effect was found on the use of *Bacillus stearothermophilus* which significantly reduced cumulative FI (SMD = −2.008, 95% CI: −3.654 to −0.362, p = 0.017) with high heterogeneity (*I*^2^ = 86.1%, *Q* = 0.001).

### Effect of fermented soybean meals on feed conversion ratio

Grand estimates obtained from SMD suggested that SBM fermentation had no effect on FCR of broiler chickens (SMD = −0.219, 95% CI: −0.501 to 0.064, p = 0.129). Restricted subgroup analysis on rearing periods also showed that FSBM did not influence FCR both in starter and finisher phase (Table 4). However, when observed on type of microorganisms used for fermentation, we found a significant decreased on FCR with either *Bacillus subtilis*+protease (SMD = −1.183, 95% CI: −1.799 to −0.567, p<0.001) or *Bacillus subtilis* alone (SMD = −1.277, 95% CI: −2.159 to −0.396, p = 0.005) with no evidence of heterogeneity from these treatments (*I*^2^ = 0.00%, *Q*>0.10). In contrast, treatment with probiotics containing LAB, yeast, and Bromelain protease significantly increased FCR of broiler chickens (SMD = 1.044, 95% CI: 0.436 to 1.652, p<0.001) with no heterogeneity (*I*^2^ = 0.00%, *Q* = 0.914).

### Meta-regression analysis

**Figure 3:**
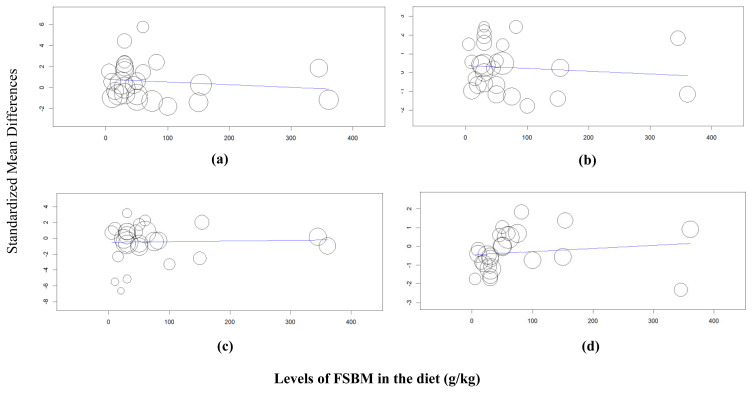
Meta-regression analysis to evaluate the relationship between fermented soybean meal (FSBM) inclusion levels in the broiler diet as predictor variable with outcome variables (a) body weight, (b) average daily gain, (c) feed intake, and (d) feed conversion ratio.

In the meta-regression analysis, we analyzed the relationship between the Hedges’ *g* effect size from the outcome variables with the inclusion levels of FSBM in the diet as predictor variable considering there were wide ranges of inclusion rates of FSBM in the broiler diet (g/kg diet, Table 1). As displayed in Figure 3, the results of the meta-regression revealed that inclusion levels had no effects on final BW (p = 0.502), ADG (p = 0.588), FI (p = 0.861), and FCR (p = 0.462) (Table 5; Figure 3).

## DISCUSSION

There are growing interests in utilizing fermented SBM as a partial or total substituting ingredient for SBM in the broiler diet due to their beneficial effects on broiler chickens’ performance. The present meta-analysis confirmed that fermented-SBM substituting diet can effectively improve final BW of broiler chickens. Results also emphasized that improvement of BW was more pronounced in the starter period (≤21 d of rearing period). This is in line with the fact that in the starter period, FI substantially increased in broiler chickens fed a diet containing FSBM (Table 4). More specifically, the discrepancies in the effect sizes were mainly influenced by the microorganisms used for fermentation because they have a different activity to degrade and utilize the SBM substrates thus may produce different fermentation products such as amino acids (AAs) and peptides [7].

Major explanations regarding beneficial effects of FSBM on broiler chickens were due to their effectiveness eliminating ANFs present in the SBM [6,7,12,14,17], as indicated in Table 3. Soybean meal is widely known to have a considerable amount of ANFs, particularly trypsin inhibitor, glycinin, and β-conglycinin that can impair the growth performance of broiler chickens especially in their early life [33]. This can be a logical reason to explain the increasing effect of utilizing FSBM in the starter phase of broiler chickens. Although there is less information on this, however, it could be speculated that feeding easily digestible materials with more nutritious material such as fermented SBM is beneficial to facilitate higher metabolism rate as well as to accommodate the immature digestive tract of broilers in this phase [34–36]. In addition, it was suggested that these ANFs also had negative effects on the immune status of broiler chickens. Elimination of glycinin and β-conglycinin in the diet can enhance the immune function of broiler chickens [6]. Several studies have demonstrated that most microbial strains used for fermentation were able to substantially remove ANFs. For instance, SBM fermented with *B. subtilis*, *B. stearothermophilus*, and *Lactobacillus* bacteria significantly reduced trypsin inhibitor by 33.6% to 100%, 74.0%, and >80%, respectively [2,7,14,37]. In addition, concentrations of glycinin, and β-conglycinin in the SBM were significantly reduced by more than 80% when fermented with *B. subtilis*+*Enterococcus faecium* and multiple strains of bacteria and yeast [6,12].

In addition, fermentation also significantly increased protein, peptide molecules, and AAs of SBM [6,7,12,13]. These nutritional improvements were associated with increasing the bioavailability of nutrients and further promoted better nutrients digestion and utilization. In regard with our findings, [9] reported that while increasing final BW, DM, CP, energy, and phosphorus digestibility were also increased. There was also evidence that supplementing FSBM in the broiler diet enhanced digestive enzyme activity [22], that may be a factor in increasing nutrient digestibility. Additionally, since small molecule peptides were produced during fermentation, it could also promote a higher nutrient absorption because small peptides can be absorbed directly and they do not require hydrolysis [38]. Furthermore, it is reasonable that the production of antimicrobial peptides created a suitable condition for intestinal flora while modulating microbial composition and improving gut integrity. Accordingly, studies reported that FSBM inclusion suppressed salmonella pathogens colonization and improved immune status and intestinal morphology [6,15,22]. In particular, Li et al [6] and Soumeh et al [11] demonstrated a reducing effect on the relative abundance of undesirable bacteria belonging to the phylum of *Proteobacteria* either with partial or total FSBM replacing SBM in broiler diets. In their study, Li et al [6] also reported increasing microbial richness as indicated by OS and Chao percentage in which *Firmicutes* phylum was predominantly presented. Abundance of Firmicutes is a good indicator for increasing non-starch carbohydrates degrading enzymes activities and it was positively associated with improvement of animal performances [39,40].

In addition to our findings, we found that strains of microorganisms performed different effects on the outcome variables including BW, ADG, FI, and FCR. In case of fermented SBM in broiler chickens, there is no evidence comparing the efficacy of microbial strains on SBM fermented products in a single study so far. However, to our knowledge, there is evidence that microbial strains have specific activity in utilizing substrates [7]. It can be explained by previous studies reporting the ability of different types of microbes in degrading ANFs and elevating nutrient compositions of FSBM [2,12–14,17]. As indicated in our results, SBM fermented with *Aspergillus oryzae* consistently increased BW, ADG, and FI of broiler chickens but had no effect on FCR. Meanwhile, *Bacillus subtilis* and *Bacillus subtilis*+protease were effective to reduce FCR although the sample sizes with these groups were small. The main factors that cause these effects were due to an increase in DM, CP, and energy retention and a decreased of indigestible fractions (ANFs) after fermentation [2,10].

Interestingly, among the large variability of FSBM inclusion levels in the diet, there was no evidence of relationship with all the outcome variables. This finding was in agreement with previous studies either with minimum FSBM use of 30 g/kg diet or with maximum inclusion of 150 g/kg diet [7,13]. This finding indicated that using low levels of FSBM aiming to improve broiler performance was sufficient. Other possible explanations may relate to the presence free of amino acids in the diet. When used in an excessive amount, free amino acids produced from fermentation of SBM concurrently increased. Since absorption of free amino acids requires higher energy than that of oligopeptides [41], increasing levels of FSBM in the diet might not contribute to further improvement in broiler chickens’ performance.

## CONCLUSION

This study suggests that broiler feed containing fermented SBM is beneficial to improve growth performance of broiler chickens, especially when it is substituted in the diet of starter period. The effects of FSBM on ADG, FI, and FCR are apparently depended on microbial strains used for fermentation. The meta-regression suggests that increasing the inclusion of FSBM did not affect broiler chickens’ performance, thus it may be used either partially or entirely to substitute the conventional SBM.

## Figures and Tables

**Table 1:** Description of the studies included in the database

No	Reference	Strain	Sex	Type of diet	Period (d)	Microbial starter	Fermentation time	Levels (g/kg diet)
1	[8]	Vencob	Mixed	Corn-SBM	42	*Aspergillus niger*	48 h	0–15
2	[22]	Ross x Ross	Male	Corn-SBM	42	*Aspergillus oryzae*	48 h	0–295
3	[9]	Ross 308	Mixed	Corn-SBM	42	*Aspergillus oryzae*		0–30
4	[2]	Cobb 500	Female	Corn-SBM	21	*Bacillus subtilis* and protease	24 h	0–150
5	[13]	Arbor Acres	n/a	Corn-SBM	42	*Bacillus Subtilis, Aspergillus niger*, and *Saccharomyces cerevisiae*	n/a	0–30
6	[16]	Arbor Acres	Mixed	Corn-SBM	35	Mixed probiotics fermented solution and bromelain	36 h	0–45
7	[10]	Ross 308	Male	Corn-SBM	35	*Bacillus subtilis*	n/a	0–30
8	[11]	Ross 308	Male	Corn-SBM	42	*Lactobacillus acidophilus*, *Lactobacillus plantarum*, *Bacillus subtilis*, and *Aspergillus oryzae*	7 d	0–345
9	[15]	Ross 308	Male	Corn-SBM	24	*Lactobacillus acidophilus*, *Lactobacillus plantarum*, *Bacillus subtilis*, and *Aspergillus oryzae*	7 d	0–361
10	[23]	Ross 308	Female	Corn-SBM	21	*Bacillus subtilis* and protease	24	0–100
11	[14]	Ross 308	n/a	Corn + Wheat and SBM	40	*Lactobacillus* (unspecific)	n/a	0–60
12	[17]	Ross 308	Mixed	Corn-SBM	42	*Bacillus subtilis*, *Lactobacillus* spp., and yeasts	n/a	0–75
13	[6]	Cobb 500	Male	Corn-SBM	36	*Bacillus amyloliquefaciens*, *Lactobacillus acidophilus*, and *Saccharomyces cerevisiae*	24 h	0–154
14	[7]	Arbor Acres	Mixed	Corn-SBM	42	*Bacillus stearothermophilus*	48 h	0–150
15	[24]	Ross 308	Mixed	Corn-SBM	35	*Bacillus velezensis Lactobacillus brevis*	36 h	0–60
16	[25]	Indian river	Mixed	Corn-SBM	35	*Saccharomyces cerevisiae*	72 h	0–40

**Table 2:** Descriptive statistics of nutrient specifications of the diets used in the meta-analysis

Nutrient specifications	n	Mean	SD	Min	Max
Starter					
ME (Kcal/kg)	36	3,002	123.46	2,796	3,200
Crude protein (%)	45	21.47	1.016	20.00	23.08
Lysine (%)	39	1.21	0.049	1.13	1.35
Methionine (%)	35	0.57	0.165	0.42	1.00
Finisher					
ME (Kcal/kg)	25	3,099	60.80	3,000	3,200
Crude protein (%)	29	19.50	0.74	18.50	20.50
Lysine (%)	23	1.03	0.031	0.97	1.07
Methionine (%)	21	0.42	0.053	0.36	0.52

Max, maximum; Min, minimum; n, number of samples; SD, standard deviation; ME, metabolizable energy.

**Table 3:** Descriptive statistics to compare chemical composition profile between soybean meal and fermented soybean meal of the studies included in the meta-analysis

Compositions	n	SBM		FSBM	
	
		Mean	SD	Mean	SD
Crude protein (g/kg DM)	21	433.9	40.27	495.9	10.89
Lys (g/kg DM)	13	28.0	1.27	29.5	1.15
Met (g/kg DM)	13	5.3	0.42	5.5	0.34
Trypsin inhibitor (mg/g)	13	238.6	234.28	0.7	0.45
Peptide (mg/g)	6	21.8	6.90	167.4	89.75
Glycinin, (mg/g)	8	85.3	27.90	15.2	4.41
β-Conglycinin (mg/g)	10	55.3	19.98	11.2	4.61

SBM, soybean meal; FSBM; fermented soybean meal; n, sample size; SD, standard of deviation; DM, dry matter.

**Table 4:** The effect size summary of meta-analysis and sub-group analyses

Outcomes	Subgroup	N	Effect size (random effect model)			SE	p-value	Heterogeneity		Egger's test
	
			SMD	C.L (Lower to Upper)				*I* ^2^	Q	
Final BW	Starter	36	0.691	0.149	1.233	0.277	0.013	84.90	0.001	0.523
	Finisher	32	0.509	0.015	1.004	0.252	0.043	82.20	0.001	
	*Aspergillus niger*	6	0.259	−0.322	0.839	0.296	0.382	0.00	0.563	
	*Aspergillus oryzae*	2	1.865	0.679	3.051	0.605	0.002	0.00	0.417	
	*Bacillus subtilis* and protease	5	6.746	2.109	11.385	2.366	0.004	92.90	0.001	
	Multistrains (LAB+yeast)	21	0.191	−0.274	0.656	0.237	0.420	68.90	0.001	
	Mixed probiotics+bromelain	6	0.614	0.029	1.198	0.298	0.040	0.00	0.871	
	*Bacillus subtilis*	2	1.638	0.711	2.566	0.473	<0.001	0.00	0.586	
	*Lactobacillus*	8	3.443	1.401	5.485	1.042	<0.001	93.10	0.001	
	*Bacillus stearothermophilus*	6	−1.657	−2.329	−0.985	0.343	<0.001	33.10	0.188	
	*Saccharomyces cerevisiae*	6	0.175	−0.292	0.643	0.239	0.462	0.00	0.700	
	*Bacillus velezensis*	6	0.097	−0.704	0.897	0.408	0.813	0.00	0.954	
	Overall	68	0.586	0.221	0.951	0.186	0.002	83.50	0.001	
	
ADG	Starter	33	0.355	−0.188	0.897	0.277	0.200	61.50	0.001	0.069
	Finisher	32	0.256	−0.199	0.711	0.232	0.180	71.10	0.001	
	*Aspergillus niger*	6	0.258	−0.322	0.838	0.296	0.383	0.00	0.564	
	*Aspergillus oryzae*	2	1.865	0.679	3.051	0.605	0.002	0.00	0.417	
	*Bacillus subtilis* and protease	2	0.192	−0.273	0.657	0.237	0.419	93.20	0.001	
	Multistrains (LAB+yeast)	21	0.192	−0.273	0.657	0.237	0.419	69.00	0.001	
	Mixed probiotics+bromelain	6	0.612	0.027	1.196	0.298	0.040	0.00	0.873	
	*Bacillus subtilis*	2	1.640	0.712	2.567	0.473	<0.001	0.00	0.585	
	*Lactobacillus*	8	0.591	0.172	1.009	0.214	0.006	0.00	0.472	
	*Bacillus stearothermophilus*	6	−1.654	−2.323	−0.985	0.341	<0.001	32.60	0.191	
	Overall	65	0.294	−0.060	0.648	0.180	0.103	66.30	0.001	
	
FI	Starter	32	−0.337	−0.879	0.205	0.277	0.223	80.30	0.001	0.109
	Finisher	31	0.582	0.037	1.128	0.278	0.036	88.90	0.001	
	*Aspergillus niger*	3	−0.067	−0.256	1.923	1.015	0.948	79.30	0.008	
	*Aspergillus oryzae*	2	2.127	0.453	3.801	0.854	0.013	42.10	0.189	
	*Bacillus subtilis* and protease	5	3.309	0.95	5.668	1.204	0.006	84.70	0.001	
	Multistrains (LAB+yeast)	21	−0.255	−0.912	0.402	0.335	0.447	81.20	0.001	
	Mixed probiotics+bromelain	6	1.681	1.016	2.346	0.339	<0.001	0.00	0.857	
	*Bacillus subtilis*	2	0.299	−0.731	1.328	0.525	0.570	36.80	0.208	
	*Lactobacillus*	6	−0.405	−3.387	2.577	1.522	0.790	93.90	0.001	
	*Bacillus stearothermophilus*	6	−2.008	−3.654	−0.362	0.84	0.017	86.10	0.001	
	Overall	63	0.115	−0.272	0.502	0.197	0.560	85.90	0.001	
	
FCR	Starter	34	−0.122	−0.539	0.295	0.213	0.567	65.30	0.001	0.422
	Finisher	32	−0.322	−0.706	0.062	0.196	0.100	60.70	0.001	
	*Aspergillus niger*	6	−606	−1.525	0.313	0.469	0.196	55.10	0.049	
	*Aspergillus oryzae*	2	−2.545	−5.72	0.63	1.62	0.116	78.80	0.030	
	*Bacillus subtilis* and protease	5	−1.183	−1.799	−0.567	0.314	<0.001	0.00	0.943	
	Multistrains (LAB+yeast)	21	0.064	−0.396	0.524	0.235	0.784	68.50	0.001	
	Mixed probiotics+bromelain	6	1.044	0.436	1.652	0.31	<0.001	0.00	0.914	
	*Bacillus subtilis*	2	−1.277	−2.159	−0.396	0.45	0.005	0.00	0.530	
	*Lactobacillus*	6	−0.502	−1.123	0.118	0.317	0.113	49.50	0.078	
	*Bacillus stearothermophilus*	6	−0.168	−0.69	0.353	0.266	0.527	17.90	0.298	
	Overall	66	−0.219	−0.501	0.064	0.144	0.129	62.90	0.001	

SMD, standardized mean differences; SE, standard error; BW, body weight; ADG, average daily gain; FI, feed intake; FCR, feed conversion ratio.

**Table 5:** Meta-regression analysis for moderator variable that influenced the effect of fermented soybean meal inclusion on broiler chickens’ performance (standardized mean differences)

Outcomes	Intercept	SE_intercept_	Slope	SE_slope_	p-value
Final body weight	0.791	0.444	−0.003	0.004	0.502
Average daily gain	0.37	0.316	−0.001	0.003	0.588
Feed intake	−0.519	0.543	0.001	0.005	0.861
Feed conversion ratio	−0.443	0.258	0.002	0.002	0.462

SE, standard of error.

## SUPPLEMENTARY MATERIAL

**Supplementary Figure S1.** Forest plot of subgroup meta-analysis of the effect of fermented-SBM on final body weight of broiler chickens based on rearing periods **Supplementary Figure S2.** Forest plot of subgroup meta-analysis based on inoculant used for fermentation of fermented-SBM on final body weight of broiler chickens **Supplementary Figure S3.** Forest plot of subgroup meta-analysis of the effect of fermented-SBM on average daily gain of broiler chickens based on rearing periods **Supplementary Figure S4.** Forest plot of subgroup meta-analysis based on inoculant used for fermentation of fermented-SBM on average daily gain of broiler chickens **Supplementary Figure S5.** Forest plot of subgroup meta-analysis of the effect of fermented-SBM on feed intake of broiler chickens based on rearing periods **Supplementary Figure S6.** Forest plot of subgroup meta-analysis based on inoculant used for fermentation of fermented-SBM on feed intake of broiler chickens **Supplementary Figure S7.** Forest plot of subgroup meta-analysis of the effect of fermented-SBM on feed conversion ratio of broiler chickens based on rearing periods **Supplementary Figure S8.** Forest plot of subgroup meta-analysis based on inoculant used for fermentation of fermented-SBM on feed conversion ratio of broiler chickens
