# Supplementary material for: Effect of feeding fermented soybean meal on broiler chickens’ performance: a meta-analysis
Source: Anim Biosci. 2022 Jun 24;35(12):1881–91. doi: 10.5713/ab.21.0546 (PMC9659450; doi:10.5713/ab.21.0546)
Supplement: Supplementary file 1 [file ab-21-0546-suppl.pdf]

Effect of feeding fermented soybean meal on broiler chickens' performance: A meta-analysis

Supplementary materials 1: Forest plots of the meta-analysis

Studies	Estimate (95% C.I.)
Mathivanan et al. 2006	0.307 (-1.087, 1.701)
Mathivanan et al. 2006-2	0.019 (-1.367, 1.405)
Mathivanan et al. 2006-3	-0.241 (-1.632, 1.149)
Feng et al. 2007	1.448 (-0.109, 3.005)
Wang et al. 2011	8.788 (5.095, 12.482)
Wang et al. 2011-2	12.680 (7.482, 17.878)
Wang et al. 2011-3	15.944 (9.466, 22.422)
Wang et al. 2012	0.546 (-0.606, 1.699)
Wang et al. 2012-2	0.913 (-0.276, 2.102)
Wang et al. 2012-3	0.847 (-0.334, 2.029)
Li et al. 2014	0.823 (-0.621, 2.266)
Li et al. 2014-2	0.494 (-0.913, 1.900)
Li et al. 2014-3	0.230 (-1.160, 1.621)
Kim et al. 2016	1.401 (0.138, 2.663)
Kim et al. 2016-2	1.690 (0.372, 3.009)
Kim et al. 2016-3	-0.290 (-1.427, 0.848)
Kim et al. 2016-4	0.193 (-0.941, 1.327)
Jazi et al. 2019	-1.358 (-2.614, -0.103)
Cheng et al. 2019	0.029 (-1.571, 1.630)
Cheng et al. 2019-2	0.427 (-1.192, 2.045)
Chachaj et al. 2019	6.704 (4.449, 8.958)
Chachaj et al. 2019-2	12.928 (8.827, 17.029)
Guo et al. 2020	0.453 (-0.608, 1.514)
Guo et al. 2020-2	0.354 (-0.702, 1.410)
Guo et al. 2020-3	-0.840 (-1.933, 0.253)
Li et al. 2020	0.849 (-0.332, 2.031)
Li et al. 2020-2	0.096 (-1.036, 1.228)
Wu et al. 2020	-1.239 (-2.474, -0.004)
Wu et al. 2020-2	-2.924 (-4.551, -1.296)
Wu et al. 2020-3	-2.726 (-4.297, -1.154)
Premathilaka et al. 2021	0.105 (-1.028, 1.237)
Premathilaka et al. 2021-2	0.279 (-0.858, 1.417)
Premathilaka et al. 2021-3	-0.140 (-1.273, 0.993)
Tsai et al. 2021	0.115 (-1.017, 1.248)
Tsai et al. 2021-2	-0.231 (-1.366, 0.905)
Tsai et al. 2021-3	0.317 (-0.821, 1.456)
**Subgroup Starter (I^2=82.01 % , P=0.000)**	**0.691 (0.149, 1.233)**
Mathivanan et al. 2006-4	1.509 (-0.062, 3.080)
Mathivanan et al. 2006-5	0.564 (-0.850, 1.977)
Mathivanan et al. 2006-6	-0.328 (-1.723, 1.067)
Feng et al. 2007-2	2.443 (0.612, 4.274)
Wang et al. 2012-4	-0.982 (-2.180, 0.216)
Wang et al. 2012-5	-0.673 (-1.836, 0.490)
Wang et al. 2012-6	-0.583 (-1.738, 0.572)
Li et al. 2014-4	0.617 (-0.801, 2.036)
Li et al. 2014-5	1.471 (-0.091, 3.034)
Li et al. 2014-6	0.237 (-1.153, 1.628)
Kim et al. 2016-5	1.917 (0.550, 3.284)
Kim et al. 2016-6	2.186 (0.756, 3.616)
Kim et al. 2016-7	0.000 (-1.132, 1.132)
Kim et al. 2016-8	1.581 (0.284, 2.877)
Soumeh et al. 2019	1.835 (0.486, 3.183)
Jazi et al. 2019-2	-1.163 (-2.387, 0.060)
Chachaj et al. 2019-3	4.405 (2.783, 6.027)
Chachaj et al. 2019-4	5.746 (3.761, 7.731)
Guo et al. 2020-4	0.421 (-0.638, 1.480)
Guo et al. 2020-5	-1.162 (-2.295, -0.030)
Guo et al. 2020-6	-1.272 (-2.421, -0.123)
Li et al. 2020-3	2.428 (0.937, 3.919)
Li et al. 2020-4	0.257 (-0.879, 1.393)
Wu et al. 2020-4	-0.666 (-1.829, 0.496)
Wu et al. 2020-5	-1.769 (-3.103, -0.434)
Wu et al. 2020-6	-1.401 (-2.664, -0.138)
Premathilaka et al. 2021-4	0.000 (-1.132, 1.132)
Premathilaka et al. 2021-5	1.095 (-0.118, 2.309)
Premathilaka et al. 2021-6	-0.168 (-1.302, 0.965)
Tsai et al. 2021-4	0.078 (-1.054, 1.210)
Tsai et al. 2021-5	-0.455 (-1.601, 0.691)
Tsai et al. 2021-6	1.053 (-0.155, 2.260)
**Subgroup Finisher (I^2=78.83 % , P=0.000)**	**0.509 (0.015, 1.004)**
**Overall (I^2=80.35 % , P=0.000)**	**0.586 (0.221, 0.951)**

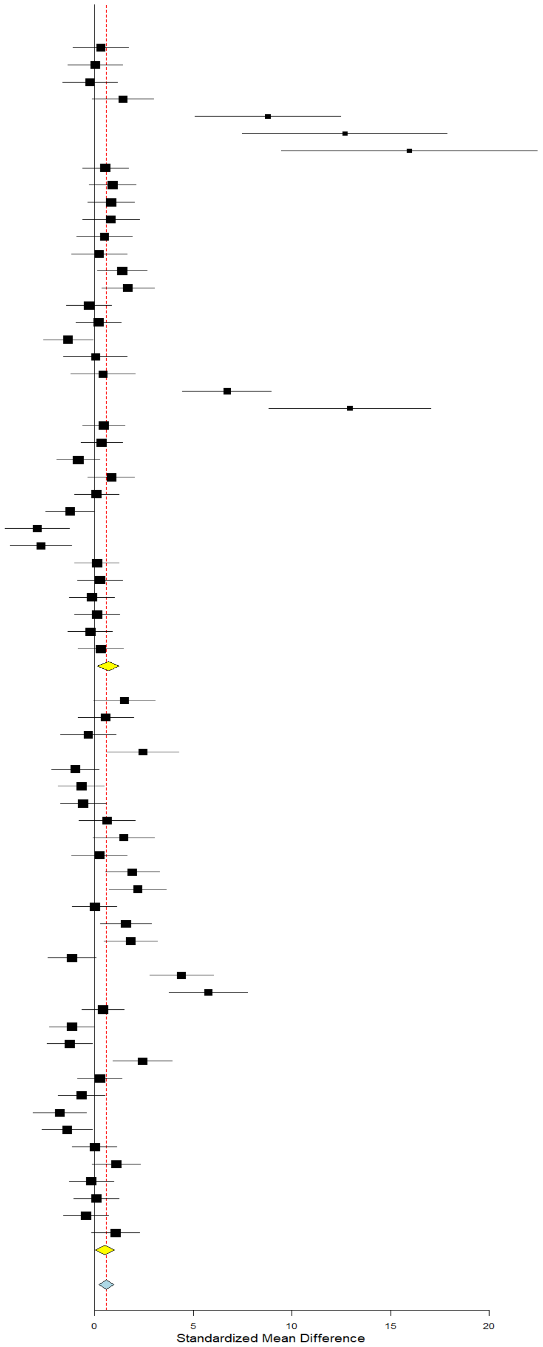

Supplementary figure 1. Forest plot of subgroup meta-analysis of the effect of fermented-SBM on final body weight of broiler chickens based on rearing periods

Studies	Estimate (95% C.I.)		
Mathivanan et al. 2006	0.307	(-1.087,	1.701)
Mathivanan et al. 2006-2	0.019	(-1.367,	1.405)
Mathivanan et al. 2006-3	-0.241	(-1.632,	1.149)
Mathivanan et al. 2006-4	1.509	(-0.062,	3.080)
Mathivanan et al. 2006-5	0.564	(-0.850,	1.977)
Mathivanan et al. 2006-6	-0.328	(-1.723,	1.067)
**Subgroup A. niger (I^2=0 % , P=0.563)**	**0.259**	**(-0.322,**	**0.839)**
Feng et al. 2007	1.448	(-0.109,	3.005)
Feng et al. 2007-2	2.443	(0.612,	4.274)
**Subgroup A. oryzae (I^2=0 % , P=0.417)**	**1.865**	**(0.679,**	**3.051)**
Wang et al. 2011	8.788	(5.095,	12.482)
Wang et al. 2011-2	12.680	(7.482,	17.878)
Wang et al. 2011-3	15.944	(9.466,	22.422)
Cheng et al. 2019	0.029	(-1.571,	1.630)
Cheng et al. 2019-2	0.427	(-1.192,	2.045)
**Subgroup B. subtillis and protease (I^2=92.9 % , P=0.000)**	**6.746**	**(2.109,**	**11.384)**
Wang et al. 2012	0.546	(-0.606,	1.699)
Wang et al. 2012-2	0.913	(-0.276,	2.102)
Wang et al. 2012-3	0.847	(-0.334,	2.029)
Wang et al. 2012-4	-0.982	(-2.180,	0.216)
Wang et al. 2012-5	-0.673	(-1.836,	0.490)
Wang et al. 2012-6	-0.583	(-1.738,	0.572)
Kim et al. 2016-2	1.690	(0.372,	3.009)
Kim et al. 2016-6	2.186	(0.756,	3.616)
Soumeh et al. 2019	1.835	(0.486,	3.183)
Jazi et al. 2019	-1.358	(-2.614,	-0.103)
Jazi et al. 2019-2	-1.163	(-2.387,	0.060)
Guo et al. 2020	0.453	(-0.608,	1.514)
Guo et al. 2020-2	0.354	(-0.702,	1.410)
Guo et al. 2020-3	-0.840	(-1.933,	0.253)
Guo et al. 2020-4	0.421	(-0.638,	1.480)
Guo et al. 2020-5	-1.162	(-2.295,	-0.030)
Guo et al. 2020-6	-1.272	(-2.421,	-0.123)
Li et al. 2020	0.849	(-0.332,	2.031)
Li et al. 2020-2	0.096	(-1.036,	1.228)
Li et al. 2020-3	2.428	(0.937,	3.919)
Li et al. 2020-4	0.257	(-0.879,	1.393)
**Subgroup Multistrains (LAB + yeast) (I^2=68.99 % , P=0.000)**	**0.191**	**(-0.274,**	**0.656)**
Li et al. 2014	0.823	(-0.621,	2.266)
Li et al. 2014-2	0.494	(-0.913,	1.900)
Li et al. 2014-3	0.230	(-1.160,	1.621)
Li et al. 2014-4	0.617	(-0.801,	2.036)
Li et al. 2014-5	1.471	(-0.091,	3.034)
Li et al. 2014-6	0.237	(-1.153,	1.628)
**Subgroup Probiotics and bromelain (I^2=0 % , P=0.871)**	**0.614**	**(0.029,**	**1.198)**
Kim et al. 2016	1.401	(0.138,	2.663)
Kim et al. 2016-5	1.917	(0.550,	3.284)
**Subgroup B. subtillis (I^2=0 % , P=0.586)**	**1.638**	**(0.711,**	**2.566)**
Kim et al. 2016-3	-0.290	(-1.427,	0.848)
Kim et al. 2016-4	0.193	(-0.941,	1.327)
Kim et al. 2016-7	0.000	(-1.132,	1.132)
Kim et al. 2016-8	1.581	(0.284,	2.877)
Chachaj et al. 2019	6.704	(4.449,	8.958)
Chachaj et al. 2019-2	12.928	(8.827,	17.029)
Chachaj et al. 2019-3	4.405	(2.783,	6.027)
Chachaj et al. 2019-4	5.746	(3.761,	7.731)
**Subgroup Lactobacillus (unspecific) (I^2=93.1 % , P=0.000)**	**3.443**	**(1.401,**	**5.485)**
Wu et al. 2020	-1.239	(-2.474,	-0.004)
Wu et al. 2020-2	-2.924	(-4.551,	-1.296)
Wu et al. 2020-3	-2.726	(-4.297,	-1.154)
Wu et al. 2020-4	-0.666	(-1.829,	0.496)
Wu et al. 2020-5	-1.769	(-3.103,	-0.434)
Wu et al. 2020-6	-1.401	(-2.664,	-0.138)
**Subgroup B. stearothermophilus (I^2=33.12 % , P=0.188)**	**-1.657**	**(-2.329,**	**-0.985)**
Premathilaka et al. 2021	0.105	(-1.028,	1.237)
Premathilaka et al. 2021-2	0.279	(-0.858,	1.417)
Premathilaka et al. 2021-3	-0.140	(-1.273,	0.993)
Premathilaka et al. 2021-4	0.000	(-1.132,	1.132)
Premathilaka et al. 2021-5	1.095	(-0.118,	2.309)
Premathilaka et al. 2021-6	-0.168	(-1.302,	0.965)
**Subgroup S. cerevisiae (I^2=0 % , P=0.700)**	**0.175**	**(-0.292,**	**0.643)**
Tsai et al. 2021	0.115	(-1.017,	1.248)
Tsai et al. 2021-4	0.078	(-1.054,	1.210)
**Subgroup B. velezensis (I^2=0 % , P=0.964)**	**0.097**	**(-0.704,**	**0.897)**
Tsai et al. 2021-2	-0.231	(-1.366,	0.905)
Tsai et al. 2021-5	-0.455	(-1.601,	0.691)
**Subgroup L. brevis (I^2=0 % , P=0.785)**	**-0.342**	**(-1.148,**	**0.465)**
Tsai et al. 2021-3	0.317	(-0.821,	1.456)
Tsai et al. 2021-6	1.053	(-0.155,	2.260)
**Subgroup B. velezensis + L. brevis (I^2=0 % , P=0.385)**	**0.663**	**(-0.165,**	**1.492)**
**Overall (I^2=80.35 % , P=0.000)**	**0.586**	**(0.221,**	**0.951)**

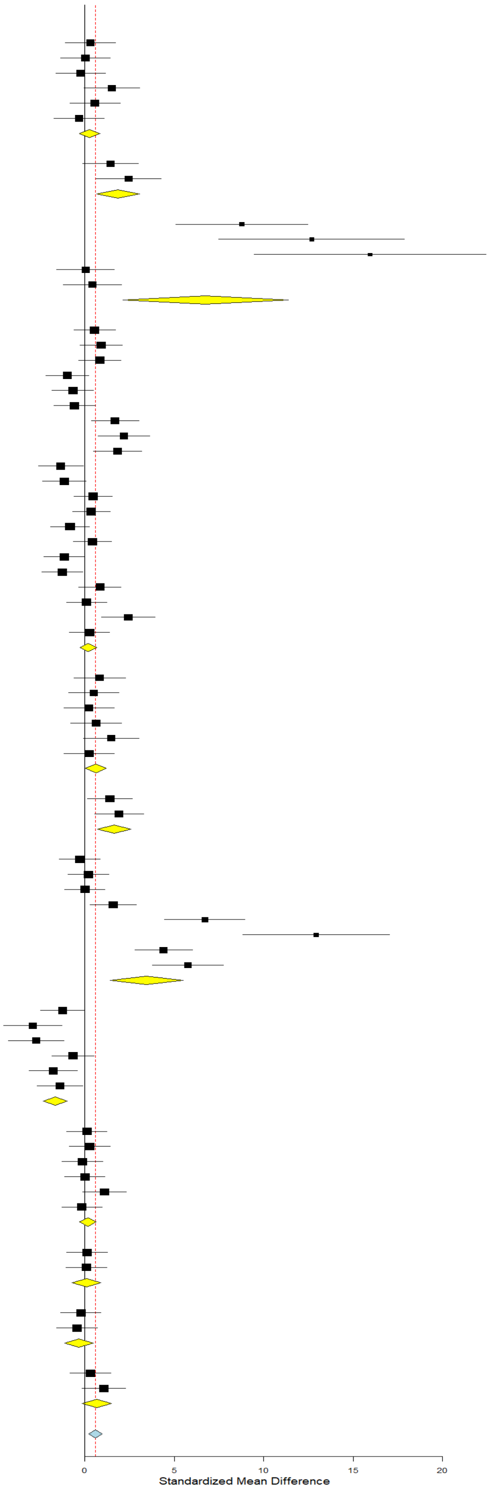

Supplementary figure 2. Forest plot of subgroup meta-analysis based on inoculant used for fermentation of fermented-SBM on final body weight of broiler chickens

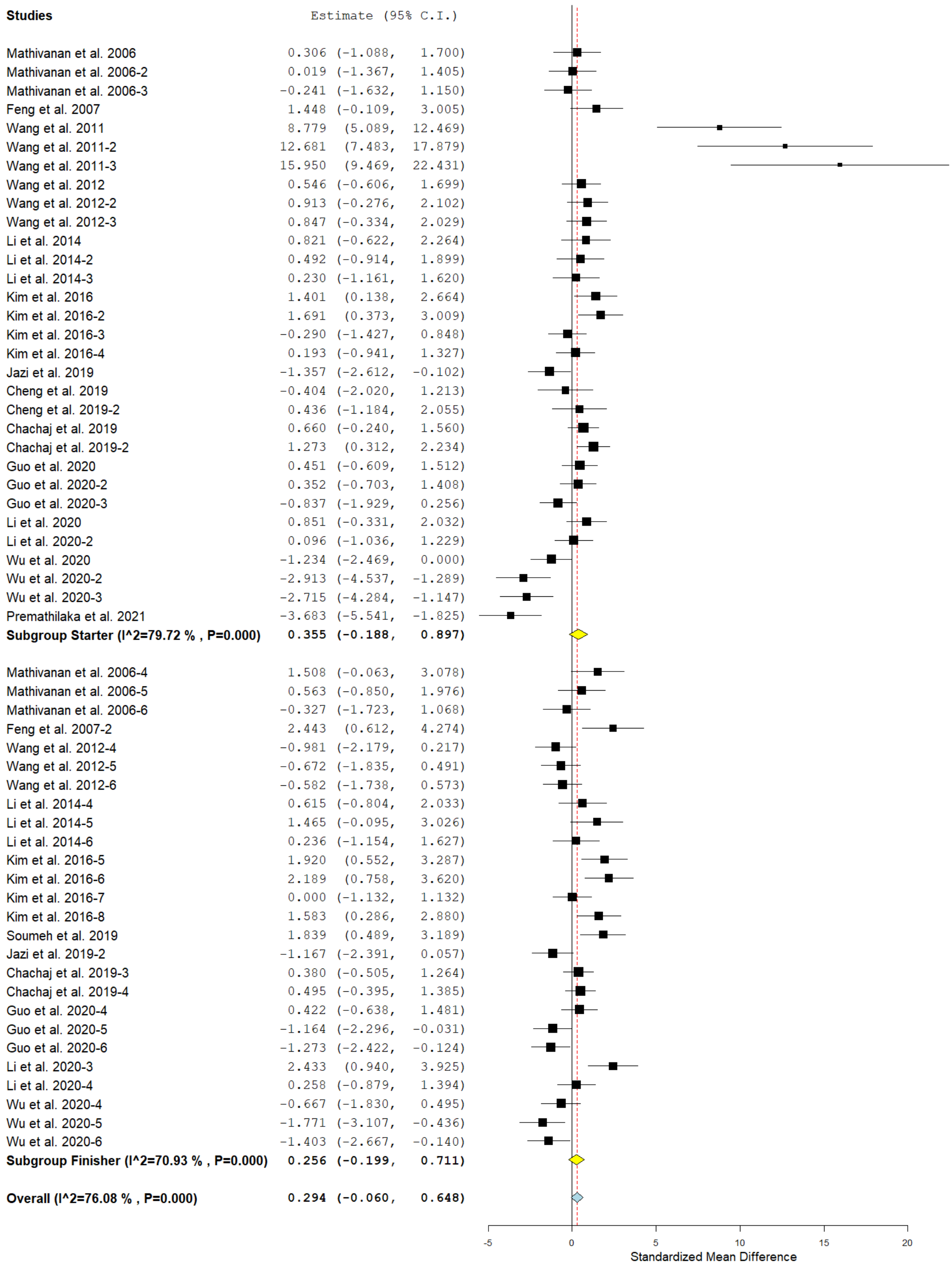

Supplementary figure 3. Forest plot of subgroup meta-analysis of the effect of fermented-SBM on average daily gain of broiler chickens based on rearing periods

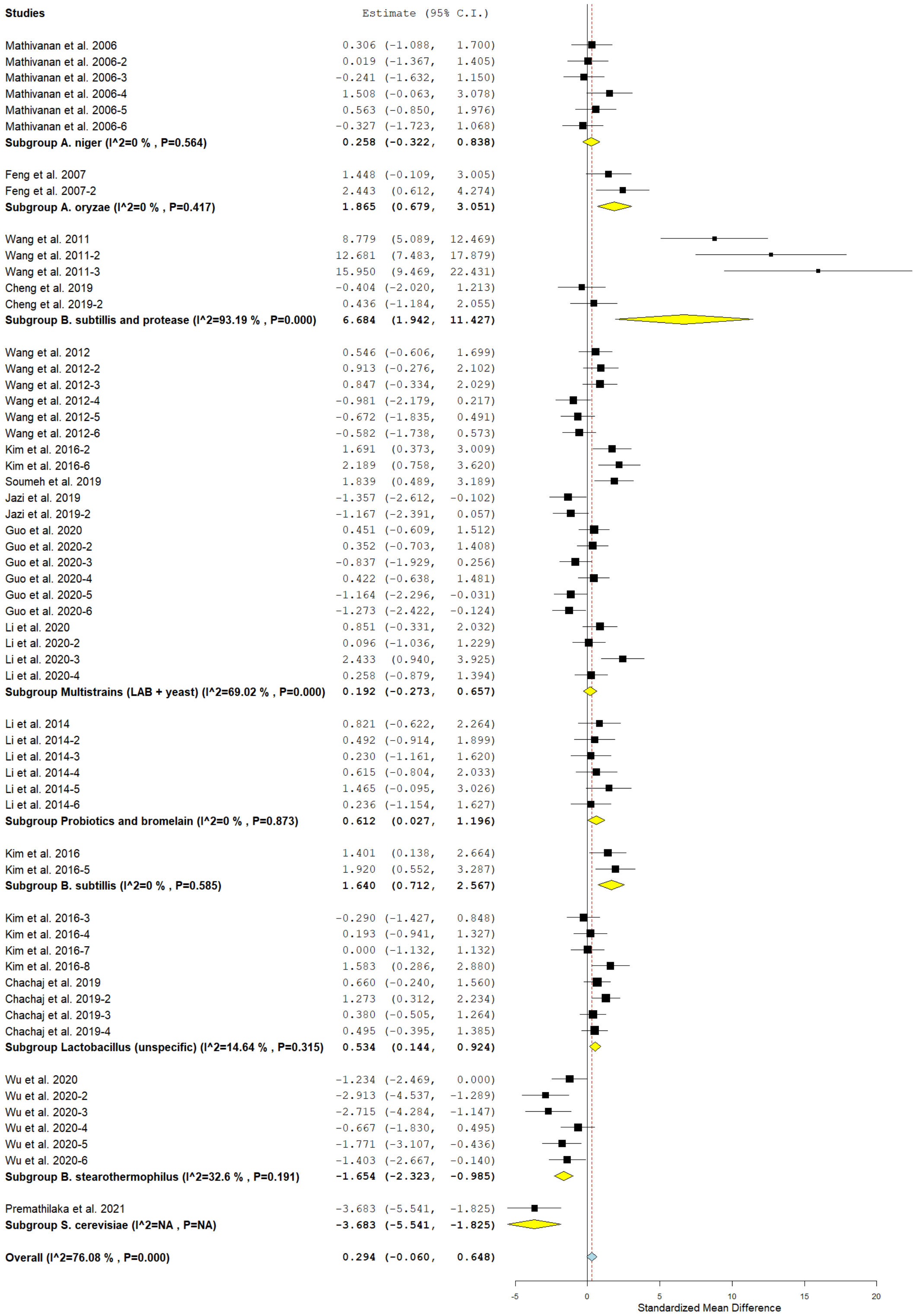

Supplementary figure 4. Forest plot of subgroup meta-analysis based on inoculant used for fermentation of fermented-SBM on average daily gain of broiler chickens

Studies	Estimate (95% C.I.)
Mathivanan et al. 2006	0.709 (-0.720, 2.138)
Mathivanan et al. 2006-2	1.206 (-0.300, 2.713)
Mathivanan et al. 2006-3	-2.292 (-4.076, -0.508)
Feng et al. 2007-2	3.185 (1.098, 5.272)
Wang et al. 2012-4	-5.481 (-7.948, -3.013)
Wang et al. 2012-5	-6.614 (-9.491, -3.736)
Wang et al. 2012-6	-5.125 (-7.467, -2.783)
Li et al. 2014-4	1.750 (0.120, 3.380)
Li et al. 2014-5	2.243 (0.474, 4.012)
Li et al. 2014-6	0.817 (-0.626, 2.259)
Kim et al. 2016-5	0.837 (-0.343, 2.017)
Kim et al. 2016-6	0.867 (-0.317, 2.051)
Kim et al. 2016-7	-0.658 (-1.820, 0.504)
Kim et al. 2016-8	0.359 (-0.782, 1.499)
Soumeh et al. 2019	0.166 (-0.968, 1.299)
Jazi et al. 2019-2	-0.958 (-2.153, 0.237)
Chachaj et al. 2019	-0.585 (-1.480, 0.310)
Chachaj et al. 2019-2	0.803 (-0.108, 1.715)
Guo et al. 2020-4	-0.094 (-1.142, 0.955)
Guo et al. 2020-5	-1.069 (-2.189, 0.051)
Guo et al. 2020-6	-0.407 (-1.465, 0.652)
Li et al. 2020-3	-0.323 (-1.462, 0.816)
Li et al. 2020-4	1.991 (0.607, 3.375)
Wu et al. 2020-4	-0.666 (-1.829, 0.496)
Wu et al. 2020-5	-3.260 (-4.986, -1.533)
Wu et al. 2020-6	-2.520 (-4.035, -1.004)
Premathilaka et al. 2021-4	0.231 (-0.905, 1.366)
Premathilaka et al. 2021-5	-0.019 (-1.151, 1.112)
Premathilaka et al. 2021-6	-0.327 (-1.466, 0.812)
**Subgroup Finisher (I^2=79.7 % , P=0.000)**	**-0.337 (-0.879, 0.205)**
Feng et al. 2007	1.403 (-0.144, 2.950)
Wang et al. 2011	3.766 (1.882, 5.651)
Wang et al. 2011-2	4.708 (2.511, 6.905)
Wang et al. 2011-3	7.376 (4.215, 10.536)
Wang et al. 2012	2.087 (0.681, 3.493)
Wang et al. 2012-2	0.669 (-0.493, 1.832)
Wang et al. 2012-3	-0.906 (-2.094, 0.282)
Li et al. 2014	1.835 (0.183, 3.487)
Li et al. 2014-2	1.957 (0.272, 3.643)
Li et al. 2014-3	1.835 (0.183, 3.487)
Kim et al. 2016	-0.214 (-1.349, 0.921)
Kim et al. 2016-2	0.171 (-0.962, 1.305)
Kim et al. 2016-3	-0.685 (-1.849, 0.479)
Kim et al. 2016-4	-0.514 (-1.664, 0.636)
Jazi et al. 2019	-0.907 (-2.095, 0.281)
Cheng et al. 2019	-0.229 (-1.834, 1.377)
Cheng et al. 2019-2	1.903 (-0.026, 3.832)
Guo et al. 2020	0.686 (-0.392, 1.764)
Guo et al. 2020-2	0.202 (-0.848, 1.252)
Guo et al. 2020-3	0.282 (-0.771, 1.334)
Li et al. 2020	1.341 (0.089, 2.593)
Li et al. 2020-2	0.377 (-0.765, 1.518)
Wu et al. 2020	1.143 (-0.077, 2.364)
Wu et al. 2020-2	-3.326 (-5.072, -1.579)
Wu et al. 2020-3	-3.942 (-5.883, -2.001)
Premathilaka et al. 2021	0.054 (-1.078, 1.186)
Premathilaka et al. 2021-2	0.285 (-0.853, 1.422)
Premathilaka et al. 2021-3	-0.149 (-1.282, 0.984)
**Subgroup Starter (I^2=78.13 % , P=0.000)**	**0.582 (0.037, 1.128)**
**Overall (I^2=79.28 % , P=0.000)**	**0.115 (-0.272, 0.502)**

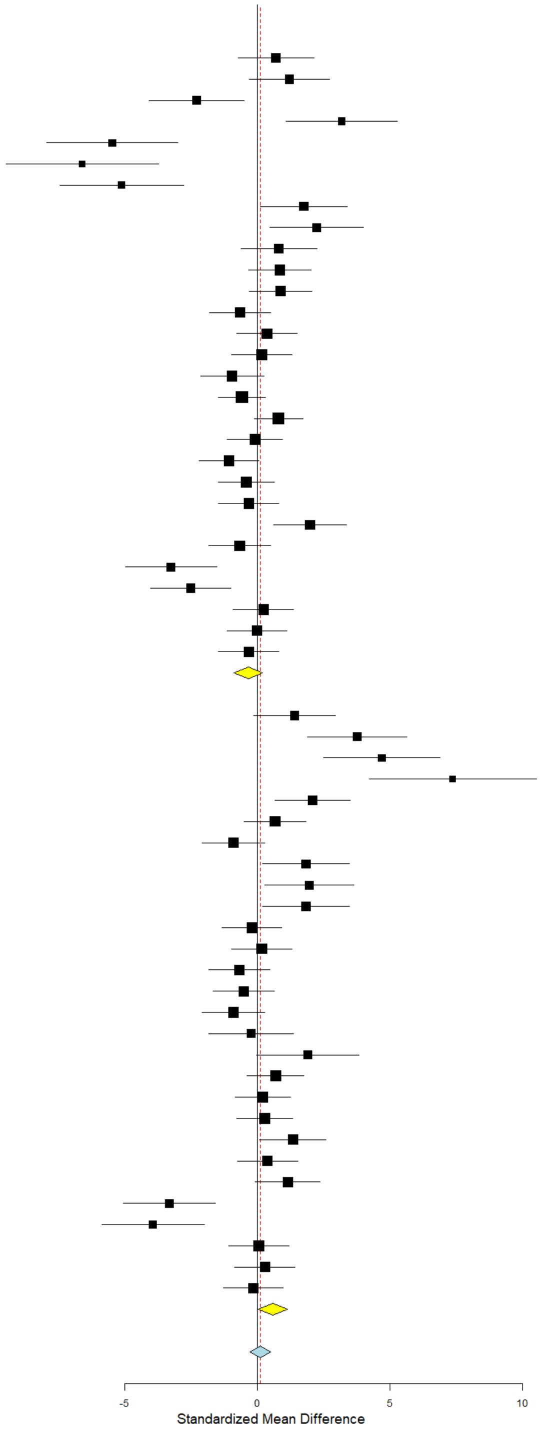

Supplementary figure 5. Forest plot of subgroup meta-analysis of the effect of fermented-SBM on feed intake of broiler chickens based on rearing periods

Studies	Estimate (95% C.I.)
Mathivanan et al. 2006	0.709 (-0.720, 2.138)
Mathivanan et al. 2006-2	1.206 (-0.300, 2.713)
Mathivanan et al. 2006-3	-2.292 (-4.076, -0.508)
**Subgroup A. niger (I^2=79.33 % , P=0.008)**	**-0.067 (-2.056, 1.923)**
Feng et al. 2007	1.403 (-0.144, 2.950)
Feng et al. 2007-2	3.185 (1.098, 5.272)
**Subgroup A. oryzae (I^2=44.66 % , P=0.179)**	**2.151 (0.427, 3.874)**
Wang et al. 2011	3.766 (1.882, 5.651)
Wang et al. 2011-2	4.708 (2.511, 6.905)
Wang et al. 2011-3	7.376 (4.215, 10.536)
Cheng et al. 2019	-0.229 (-1.834, 1.377)
Cheng et al. 2019-2	1.903 (-0.026, 3.832)
**Subgroup B. subtillis and protease (I^2=84.73 % , P=0.000)**	**3.309 (0.950, 5.668)**
Wang et al. 2012	2.087 (0.681, 3.493)
Wang et al. 2012-2	0.669 (-0.493, 1.832)
Wang et al. 2012-3	-0.906 (-2.094, 0.282)
Wang et al. 2012-4	-5.481 (-7.948, -3.013)
Wang et al. 2012-5	-6.614 (-9.491, -3.736)
Wang et al. 2012-6	-5.125 (-7.467, -2.783)
Kim et al. 2016-2	0.171 (-0.962, 1.305)
Kim et al. 2016-6	0.867 (-0.317, 2.051)
Soumeh et al. 2019	0.166 (-0.968, 1.299)
Jazi et al. 2019	-0.907 (-2.095, 0.281)
Jazi et al. 2019-2	-0.958 (-2.153, 0.237)
Guo et al. 2020	0.686 (-0.392, 1.764)
Guo et al. 2020-2	0.202 (-0.848, 1.252)
Guo et al. 2020-3	0.282 (-0.771, 1.334)
Guo et al. 2020-4	-0.094 (-1.142, 0.955)
Guo et al. 2020-5	-1.069 (-2.189, 0.051)
Guo et al. 2020-6	-0.407 (-1.465, 0.652)
Li et al. 2020	1.341 (0.089, 2.593)
Li et al. 2020-2	0.377 (-0.765, 1.518)
Li et al. 2020-3	-0.323 (-1.462, 0.816)
Li et al. 2020-4	1.991 (0.607, 3.375)
**Subgroup Multistrains (LAB + yeast) (I^2=79.09 % , P=0.000)**	**-0.304 (-0.901, 0.293)**
Li et al. 2014	1.835 (0.183, 3.487)
Li et al. 2014-2	1.957 (0.272, 3.643)
Li et al. 2014-3	1.835 (0.183, 3.487)
Li et al. 2014-4	1.750 (0.120, 3.380)
Li et al. 2014-5	2.243 (0.474, 4.012)
Li et al. 2014-6	0.817 (-0.626, 2.259)
**Subgroup Mixed probiotics fermented solution and bromelain (I^2=0 % , P=0.857)**	**1.681 (1.016, 2.346)**
Kim et al. 2016	-0.214 (-1.349, 0.921)
Kim et al. 2016-5	0.837 (-0.343, 2.017)
**Subgroup B. subtillis (I^2=36.85 % , P=0.208)**	**0.299 (-0.731, 1.328)**
Kim et al. 2016-3	-0.685 (-1.849, 0.479)
Kim et al. 2016-4	-0.514 (-1.664, 0.636)
Kim et al. 2016-7	-0.658 (-1.820, 0.504)
Kim et al. 2016-8	0.359 (-0.782, 1.499)
Chachaj et al. 2019	-0.585 (-1.480, 0.310)
Chachaj et al. 2019-2	0.803 (-0.108, 1.715)
**Subgroup Lactobacillus (unspecific) (I^2=35.93 % , P=0.167)**	**-0.179 (-0.719, 0.361)**
Wu et al. 2020	1.143 (-0.077, 2.364)
Wu et al. 2020-2	-3.326 (-5.072, -1.579)
Wu et al. 2020-3	-3.942 (-5.883, -2.001)
Wu et al. 2020-4	-0.666 (-1.829, 0.496)
Wu et al. 2020-5	-3.260 (-4.986, -1.533)
Wu et al. 2020-6	-2.520 (-4.035, -1.004)
**Subgroup B. stearothermophilus (I^2=86.1 % , P=0.000)**	**-2.008 (-3.654, -0.362)**
Premathilaka et al. 2021	0.054 (-1.078, 1.186)
Premathilaka et al. 2021-2	0.285 (-0.853, 1.422)
Premathilaka et al. 2021-3	-0.149 (-1.282, 0.984)
Premathilaka et al. 2021-4	0.231 (-0.905, 1.366)
Premathilaka et al. 2021-5	-0.019 (-1.151, 1.112)
Premathilaka et al. 2021-6	-0.327 (-1.466, 0.812)
**Subgroup S. cerevisiae (I^2=0 % , P=0.978)**	**0.013 (-0.451, 0.476)**
**Overall (I^2=79.28 % , P=0.000)**	**0.115 (-0.272, 0.502)**

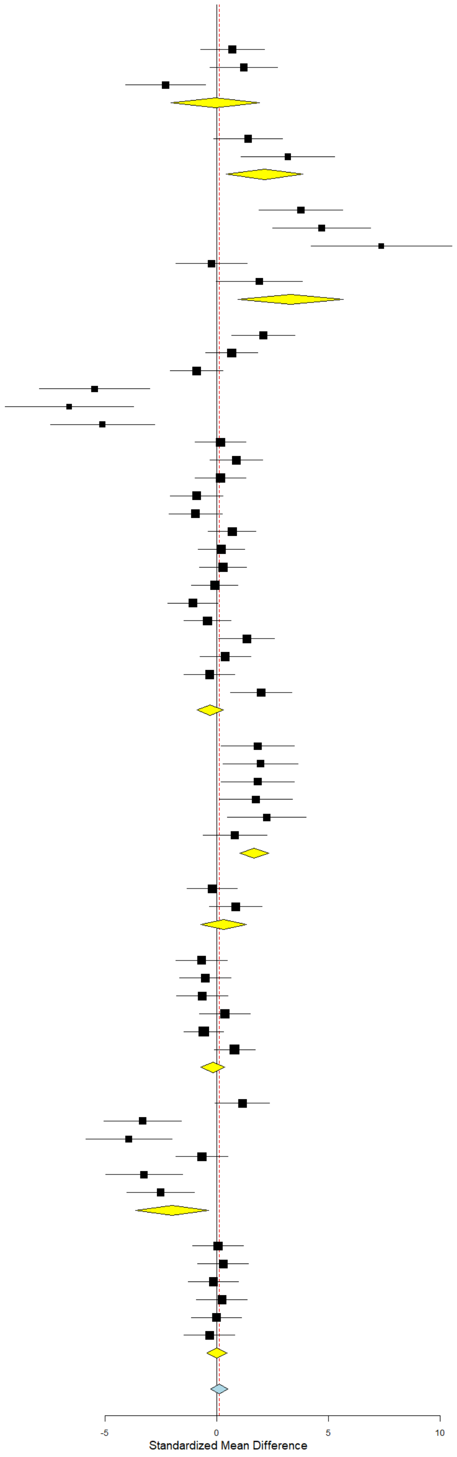

Supplementary figure 6. Forest plot of subgroup meta-analysis based on inoculant used for fermentation of fermented-SBM on feed intake of broiler chickens

Studies	Estimate (95% C.I.)
Mathivanan et al. 2006	-2.461 (-4.298, -0.624)
Mathivanan et al. 2006-2	0.290 (-1.104, 1.683)
Mathivanan et al. 2006-3	0.724 (-0.707, 2.154)
Feng et al. 2007	-4.343 (-6.883, -1.804)
Wang et al. 2011	-0.923 (-2.113, 0.268)
Wang et al. 2011-2	-1.476 (-2.753, -0.200)
Wang et al. 2011-3	-1.107 (-2.323, 0.108)
Wang et al. 2012	0.738 (-0.431, 1.908)
Wang et al. 2012-2	-0.554 (-1.707, 0.599)
Wang et al. 2012-3	-1.476 (-2.753, -0.200)
Li et al. 2014	1.025 (-0.449, 2.499)
Li et al. 2014-2	1.581 (-0.007, 3.169)
Li et al. 2014-3	1.564 (-0.020, 3.147)
Kim et al. 2016	-1.015 (-2.217, 0.187)
Kim et al. 2016-2	-1.015 (-2.217, 0.187)
Kim et al. 2016-3	-0.092 (-1.224, 1.040)
Kim et al. 2016-4	-0.369 (-1.510, 0.772)
Jazi et al. 2019	1.845 (0.494, 3.197)
Cheng et al. 2019	-1.682 (-3.544, 0.180)
Cheng et al. 2019-2	-0.931 (-2.616, 0.754)
Guo et al. 2020	0.312 (-0.742, 1.366)
Guo et al. 2020-2	0.000 (-1.048, 1.048)
Guo et al. 2020-3	1.560 (0.363, 2.756)
Li et al. 2020	0.923 (-0.268, 2.113)
Li et al. 2020-2	0.461 (-0.685, 1.608)
Wu et al. 2020	1.107 (-0.108, 2.323)
Wu et al. 2020-2	-0.185 (-1.319, 0.949)
Wu et al. 2020-3	-0.554 (-1.707, 0.599)
**Subgroup Starter (I^2=65.33 % , P=0.000)**	**-0.122 (-0.539, 0.295)**
Mathivanan et al. 2006-4	-1.737 (-3.364, -0.111)
Mathivanan et al. 2006-5	-0.145 (-1.532, 1.243)
Mathivanan et al. 2006-6	-0.869 (-2.318, 0.581)
Feng et al. 2007-2	-1.086 (-2.570, 0.399)
Wang et al. 2012-4	-0.395 (-1.538, 0.747)
Wang et al. 2012-5	-0.923 (-2.113, 0.268)
Wang et al. 2012-6	-0.659 (-1.821, 0.503)
Li et al. 2014-4	1.028 (-0.447, 2.502)
Li et al. 2014-5	0.680 (-0.745, 2.106)
Li et al. 2014-6	0.594 (-0.823, 2.010)
Kim et al. 2016-5	-1.582 (-2.878, -0.285)
Kim et al. 2016-6	-1.714 (-3.037, -0.391)
Kim et al. 2016-7	-0.527 (-1.678, 0.624)
Kim et al. 2016-8	-1.582 (-2.878, -0.285)
Soumeh et al. 2019	-2.307 (-3.767, -0.847)
Jazi et al. 2019-2	0.923 (-0.268, 2.113)
Chachaj et al. 2019	-1.197 (-2.149, -0.245)
Chachaj et al. 2019-2	0.479 (-0.410, 1.368)
Guo et al. 2020-4	-0.468 (-1.530, 0.594)
Guo et al. 2020-5	0.000 (-1.048, 1.048)
Guo et al. 2020-6	0.702 (-0.377, 1.781)
Li et al. 2020-3	1.845 (0.494, 3.197)
Li et al. 2020-4	1.384 (0.124, 2.644)
Wu et al. 2020-4	0.000 (-1.132, 1.132)
Wu et al. 2020-5	-0.738 (-1.908, 0.431)
Wu et al. 2020-6	-0.554 (-1.707, 0.599)
**Subgroup Finisher (I^2=60.69 % , P=0.000)**	**-0.322 (-0.706, 0.062)**
**Overall (I^2=62.98 % , P=0.000)**	**-0.219 (-0.501, 0.064)**

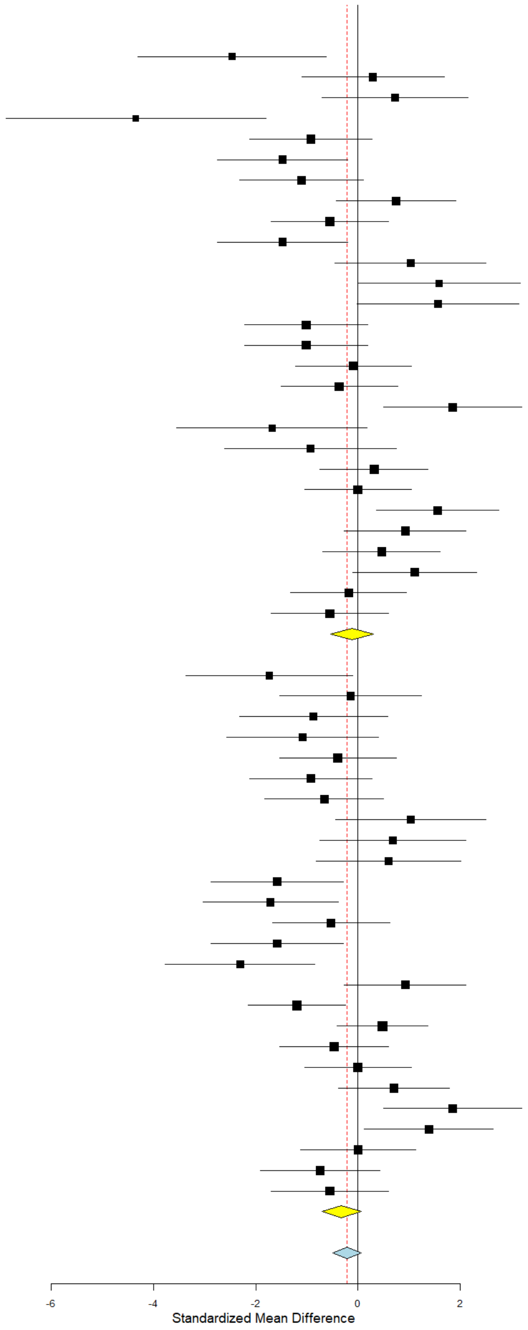

Supplementary figure 7. Forest plot of subgroup meta-analysis of the effect of fermented-SBM on feed conversion ratio of broiler chickens based on rearing periods

Studies	Estimate (95% C.I.)
Mathivanan et al. 2006	-2.461 (-4.298, -0.624)
Mathivanan et al. 2006-2	0.290 (-1.104, 1.683)
Mathivanan et al. 2006-3	0.724 (-0.707, 2.154)
Mathivanan et al. 2006-4	-1.737 (-3.364, -0.111)
Mathivanan et al. 2006-5	-0.145 (-1.532, 1.243)
Mathivanan et al. 2006-6	-0.869 (-2.318, 0.581)
**Subgroup Aspergillus niger (I^2=55.13 % , P=0.049)**	**-0.606 (-1.525, 0.313)**
Feng et al. 2007	-4.343 (-6.883, -1.804)
Feng et al. 2007-2	-1.086 (-2.570, 0.399)
**Subgroup Aspergillus oryzae (I^2=78.77 % , P=0.030)**	**-2.545 (-5.720, 0.630)**
Wang et al. 2011	-0.923 (-2.113, 0.268)
Wang et al. 2011-2	-1.476 (-2.753, -0.200)
Wang et al. 2011-3	-1.107 (-2.323, 0.108)
Cheng et al. 2019	-1.682 (-3.544, 0.180)
Cheng et al. 2019-2	-0.931 (-2.616, 0.754)
**Subgroup Bacillus subtillis and protease (I^2=0 % , P=0.943)**	**-1.183 (-1.799, -0.567)**
Wang et al. 2012	0.738 (-0.431, 1.908)
Wang et al. 2012-2	-0.554 (-1.707, 0.599)
Wang et al. 2012-3	-1.476 (-2.753, -0.200)
Wang et al. 2012-4	-0.395 (-1.538, 0.747)
Wang et al. 2012-5	-0.923 (-2.113, 0.268)
Wang et al. 2012-6	-0.659 (-1.821, 0.503)
Kim et al. 2016-2	-1.015 (-2.217, 0.187)
Kim et al. 2016-6	-1.714 (-3.037, -0.391)
Soumeh et al. 2019	-2.307 (-3.767, -0.847)
Jazi et al. 2019	1.845 (0.494, 3.197)
Jazi et al. 2019-2	0.923 (-0.268, 2.113)
Guo et al. 2020	0.312 (-0.742, 1.366)
Guo et al. 2020-2	0.000 (-1.048, 1.048)
Guo et al. 2020-3	1.560 (0.363, 2.756)
Guo et al. 2020-4	-0.468 (-1.530, 0.594)
Guo et al. 2020-5	0.000 (-1.048, 1.048)
Guo et al. 2020-6	0.702 (-0.377, 1.781)
Li et al. 2020	0.923 (-0.268, 2.113)
Li et al. 2020-2	0.461 (-0.685, 1.608)
Li et al. 2020-3	1.845 (0.494, 3.197)
Li et al. 2020-4	1.384 (0.124, 2.644)
**Subgroup Multistrains (LAB + yeast) (I^2=68.48 % , P=0.000)**	**0.064 (-0.396, 0.524)**
Li et al. 2014	1.025 (-0.449, 2.499)
Li et al. 2014-2	1.581 (-0.007, 3.169)
Li et al. 2014-3	1.564 (-0.020, 3.147)
Li et al. 2014-4	1.028 (-0.447, 2.502)
Li et al. 2014-5	0.680 (-0.745, 2.106)
Li et al. 2014-6	0.594 (-0.823, 2.010)
**Subgroup Mixed probiotics fermented solution and bromelain (I^2=0 % , P=0.914)**	**1.044 (0.436, 1.652)**
Kim et al. 2016	-1.015 (-2.217, 0.187)
Kim et al. 2016-5	-1.582 (-2.878, -0.285)
**Subgroup Bacillus subtillis (I^2=0 % , P=0.530)**	**-1.277 (-2.159, -0.396)**
Kim et al. 2016-3	-0.092 (-1.224, 1.040)
Kim et al. 2016-4	-0.369 (-1.510, 0.772)
Kim et al. 2016-7	-0.527 (-1.678, 0.624)
Kim et al. 2016-8	-1.582 (-2.878, -0.285)
Chachaj et al. 2019	-1.197 (-2.149, -0.245)
Chachaj et al. 2019-2	0.479 (-0.410, 1.368)
**Subgroup Lactobacillus (unspecific) (I^2=49.53 % , P=0.078)**	**-0.502 (-1.123, 0.118)**
Wu et al. 2020	1.107 (-0.108, 2.323)
Wu et al. 2020-2	-0.185 (-1.319, 0.949)
Wu et al. 2020-3	-0.554 (-1.707, 0.599)
Wu et al. 2020-4	0.000 (-1.132, 1.132)
Wu et al. 2020-5	-0.738 (-1.908, 0.431)
Wu et al. 2020-6	-0.554 (-1.707, 0.599)
**Subgroup Bacillus stearothermophilus (I^2=17.88 % , P=0.298)**	**-0.168 (-0.690, 0.353)**
**Overall (I^2=62.98 % , P=0.000)**	**-0.219 (-0.501, 0.064)**

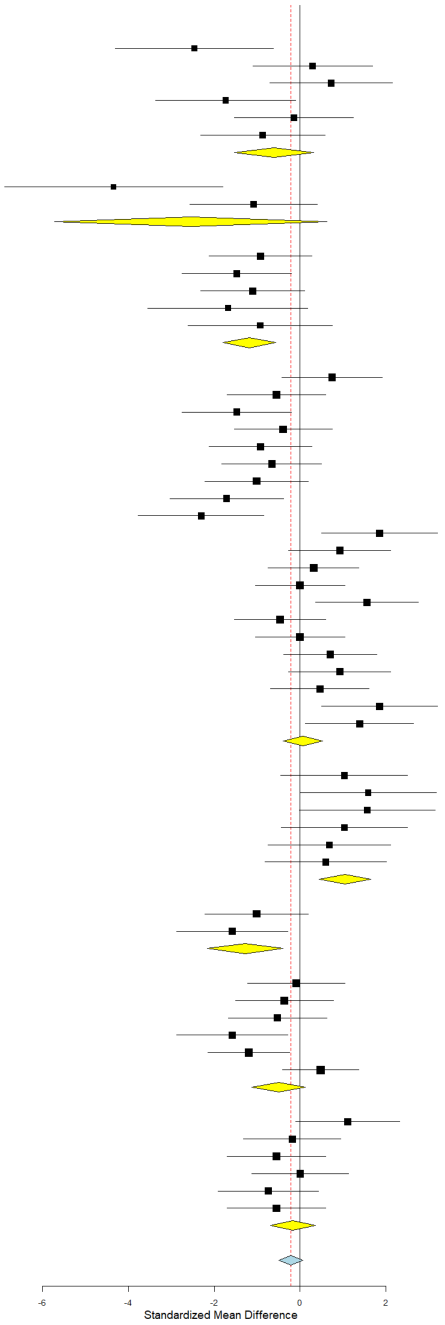

Supplementary figure 8. Forest plot of subgroup meta-analysis based on inoculant used for fermentation of fermented-SBM on feed conversion ratio of broiler chickens
